# The Placental Transcriptome in Late Gestational Hypoxia Resulting in Murine Intrauterine Growth Restriction Parallels Increased Risk of Adult Cardiometabolic Disease

**DOI:** 10.1038/s41598-018-37627-y

**Published:** 2019-02-04

**Authors:** Alison Chu, David Casero, Shanthie Thamotharan, Madhuri Wadehra, Amy Cosi, Sherin U. Devaskar

**Affiliations:** 1 David Geffen School of Medicine at UCLA, Department of Pediatrics, Division of Neonatology & Developmental Biology, Neonatal Research Center of the UCLA Children’s Discovery and Innovation Institute, 10833 Le Conte Avenue, MDCC B2-375, Los Angeles, CA 90095 USA; 2 David Geffen School of Medicine at UCLA, Department of Pathology and Laboratory Medicine, 3000 Terasaki Life Sciences Building, 610 Charles Young Drive East, Los Angeles, CA 90095 USA; 3 David Geffen School of Medicine at UCLA, Department of Pathology and Laboratory Medicine, 4525 MacDonald Research Laboratories, Los Angeles, CA 90095 USA

## Abstract

Intrauterine growth restriction (IUGR) enhances risk for adult onset cardiovascular disease (CVD). The mechanisms underlying IUGR are poorly understood, though inadequate blood flow and oxygen/nutrient provision are considered common endpoints. Based on evidence in humans linking IUGR to adult CVD, we hypothesized that in murine pregnancy, maternal late gestational hypoxia (LG-H) exposure resulting in IUGR would result in (1) placental transcriptome changes linked to risk for later CVD, and 2) adult phenotypes of CVD in the IUGR offspring. After subjecting pregnant mice to hypoxia (10.5% oxygen) from gestational day (GD) 14.5 to 18.5, we undertook RNA sequencing from GD19 placentas. Functional analysis suggested multiple changes in structural and functional genes important for placental health and function, with maximal dysregulation involving vascular and nutrient transport pathways. Concordantly, a ~10% decrease in birthweights and ~30% decrease in litter size was observed, supportive of placental insufficiency. We also found that the LG-H IUGR offspring exhibit increased risk for CVD at 4 months of age, manifesting as hypertension, increased abdominal fat, elevated leptin and total cholesterol concentrations. In summary, this animal model of IUGR links the placental transcriptional response to the stressor of gestational hypoxia to increased risk of developing cardiometabolic disease.

## Introduction

Placental insufficiency is a poorly understood complication of pregnancy, affecting up to 10% of pregnancies. It encompasses a myriad of clinical outcomes, including recurrent miscarriage, preterm birth, preeclampsia and intrauterine growth restriction (IUGR). The pathophysiology of placental insufficiency remains poorly understood, but leads to a common result - poor blood flow, and therefore, inadequate nutrient and oxygen provision to the fetus^1^. Disorders of placental insufficiency pose risks to the fetus and neonate but also have been linked to adverse long-term effects on the offspring^2–4^. Therefore, it is critical to understand the immediate adverse effects of gestational hypoxia on the key organ supporting fetal growth and development, the placenta, as well as the long-term implications on developmental programming of cardiometabolic disease.

Placental blood flow is a key regulator of nutrient and oxygen provision to the growing fetus. In human disease, altered placental vascularization has been linked as a physiologic component of placental insufficiency contributing to obstetric disorders such as preeclampsia and IUGR^5,6^. Existing *in vivo* models using various gestational stressors such as maternal food restriction, hypoxia exposure, or uterine artery ligation can all result in a phenotype of placental insufficiency and associated IUGR^7–9^. However, while the food restriction model preferentially affects nutrient transfer culminating in a chronic reduction in uteroplacental blood flow^10^, the uterine artery ligation model results in abrupt reduction in placental blood flow during late pregnancy. Both these models cause IUGR but potentially may affect different processes than those with chronic gestational hypoxia. Gestational hypoxia has been shown to affect placental structure and function, in a manner dependent on the onset, duration/chronicity, and severity of hypoxia exposure^9^. Murine models of placental insufficiency secondary to gestational hypoxia exposure have been reported to affect fetal cardiovascular development and programming of adult cardiometabolic disease by affecting inflammation and fat deposition^11^, as well as endothelial function and hemodynamics^12^. Maternal food restriction models have been well described as having effects on maternal nutrient transfer across the placenta^10,13^ and also preferentially affecting long-term endocrine and metabolic adaptations^14–17^. Therefore, while poor blood flow, inadequate nutrient provision and hypoxia all contribute towards placental insufficiency as it affects fetal outcomes, it remains important to individually dissect the effect of each of those components both on the placenta as it determines perinatal outcomes, as well as the long term effects on the offspring.

While the association between placental insufficiency exposure in utero resulting in low birth weight or prematurity and risk of adult cardiometabolic disease is well established in the human^18^, the causative factors and pathophysiology remain largely unknown. Involvement of endothelin, the renin-angiotensin system, and the sympathetic nervous system have all been implicated in the pathophysiology of adult CVD in animal studies^19^. Recent murine studies have linked gestational hypoxia to programming of adult cardiometabolic disease^11,12^, specifically with phenotypes of adiposity, altered cholesterol levels, insulin resistance, inflammation, and endothelial dysfunction, as well as renal disease^20,21^. However, these adult outcomes have not specifically been linked to the placental transcriptome. The advantage of examining the placenta is the future possibility of utilizing it as a biomarker of later disease development, as it shares both the genetic make-up and has resided in the same *in-utero* environment as the fetus. Therefore, in human pregnancy, examining the placenta, a tissue that is often discarded post-parturient, allows for interrogation of fetal responses to in utero stressors without having to invasively obtain blood or tissue from the offspring. Therefore, our specific research objectives were to evaluate: (1) the effects of gestational hypoxia on the pre-parturient placental transcriptome as specifically related to vascular function, metabolic pathways, and inflammation, and (2) phenotypes of cardiometabolic disease in hypoxia-exposed offspring, thereby providing a foundation for molecular mechanisms by which intra-uterine stressors can be linked to adult cardiometabolic disease.

## Results

### Placenta weight, birth weight, and litter sizes

There was no difference in placental weight (p = 0.90 by Student’s t-test), fetal weight (p = 0.62 by Student’s t-test) or litter size (p = 0.40 by Mann-Whitney U test) at GD19 between LG-RA (mice exposed to FiO_2_ 0.21 during late gestation, days 14.5–18.5) and LG-H (mice exposed to FiO_2_ 0.105 during late gestation, days 14.5–18.5) groups (Table 1). In our LG-H group, we did not observe a difference in fetal weights (males: 1.21 ± 0.13 g; females: 1.13 ± 0.11 g; p = 0.15 by Student’s t-test) or placental weights (males: 0.13 ± 0.04 g; females: 0.10 ± 0.04 g; p = 0.07 by Student’s t-test) by sex (n = 7–15/sex/group).

**Table 1:** Fetal/placental weight and litter size at GD19 in LG-RA and LG-H groups.

Fetal/placental weights and litter size at GD19	LG-RA (n = 5, average per litter)	LG-H (n = 4, average per litter)
Placental weight (g)	0.107 ± 0.025	0.117 ± 0.036
Fetal weights (g)	1.17 ± 0.012	1.16 ± 0.04
Litter size (# of pups per litter)	8 (5.244–8.756)	8.5 (4.97–11.53)

Data are expressed as mean ± SD for placental and fetal weights, and as median with 95% confidence intervals for litter size.

**Figure 1:**
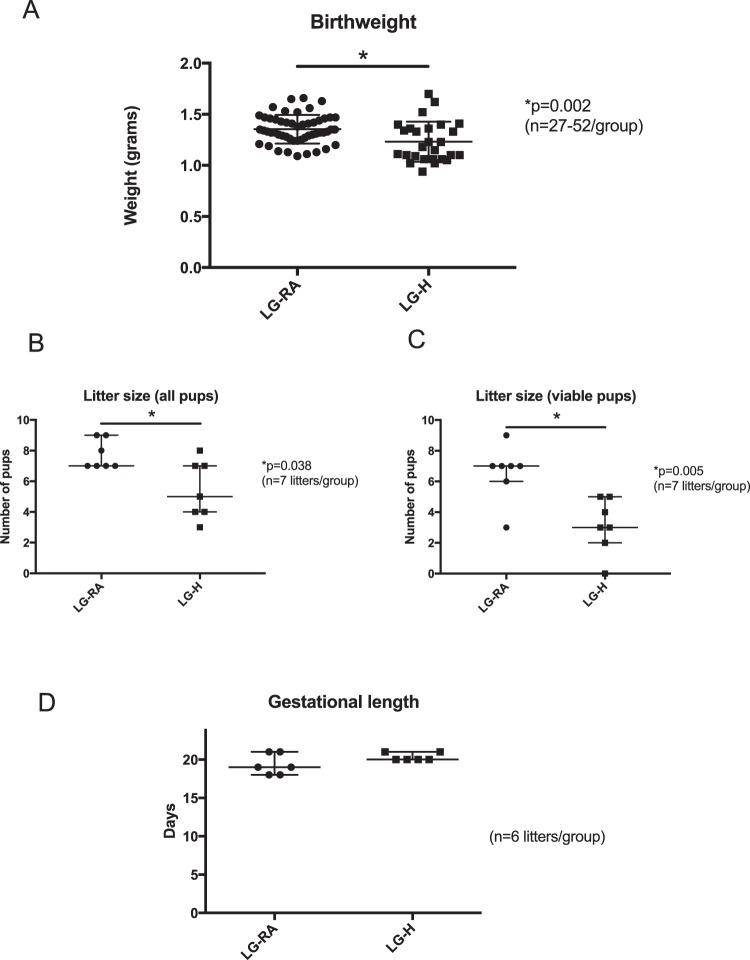
Birthweights, litter sizes, and gestational length of late gestational-room air (LG-RA) and late-gestational hypoxia (LG-H) groups. (**A**) LG-H exposure results in a ~10% decrease in birthweight compared to LG-RA. Data are represented in graphs as means ± SD. The asterisk indicates a significant difference between LG-H (squares) and LG-RA (circles) groups (p = 0.002 by Student’s t-test). (**B**) LG-H exposure results in a ~30–40% reduction in litter sizes compared to LG-RA when considering all pups delivered (viable and non-viable) (p = 0.038). Data are represented in graphs as median with 95% confidence intervals. Asterisks indicate a significant difference between indicated LG-H group compared to LG-RA by Mann-Whitney testing. (**C**) LG-H exposure results in a ~50% reduction in litter sizes compared to LG-RA when considering only viable pups (p = 0.005). Data are represented in graphs as median with 95% confidence intervals. Asterisks indicate a significant difference between indicated LG-H group compared to LG-RA by Mann-Whitney testing. (**D**) There is no difference in gestational length between LG-RA and LG-H groups (p = 0.21 by Mann-Whitney test). Data are represented in graphs as median with 95% confidence intervals.

In contrast to the placenta, LG-H mice (n = 21) demonstrated an almost 10% decrease in birthweight compared to LG-RA mice (p = 0.002 by Student’s t-test) (Fig. 1A). When comparing litter sizes, the LG-H group had a significant decrease in litter size compared to the LG-RA group, when considering both viable and non-viable pups (p = 0.038 by Mann-Whitney U test) (Fig. 1B), and when considering viable pups only (p = 0.005 by Mann-Whitney U test) (Fig. 1C). There was not a difference in gestational length between the LG-RA and LG-H group (p = 0.21 by Mann-Whitney U test) (Fig. 1D). Given the significant differences in litter sizes and the known effect of litter size on birth weight, we calculated a “corrected” birth weight per litter, by dividing the total litter weight by the average control litter size at birth (mean litter size = 7.43 pups/litter). When adjusting for the difference in litter size in this manner, there is an almost 35% decrease in birthweight (LG-RA, n = 7 litters: median 1.387 g, 95% CI: 1.194–1.512 g; LG-H, n = 5 litters: median 0.817 g, 95% CI: 0.520–1.271 g; p = 0.018 by Mann-Whitney U test).

### Placental gene expression changes induced by late gestational hypoxia

**Figure 2:**
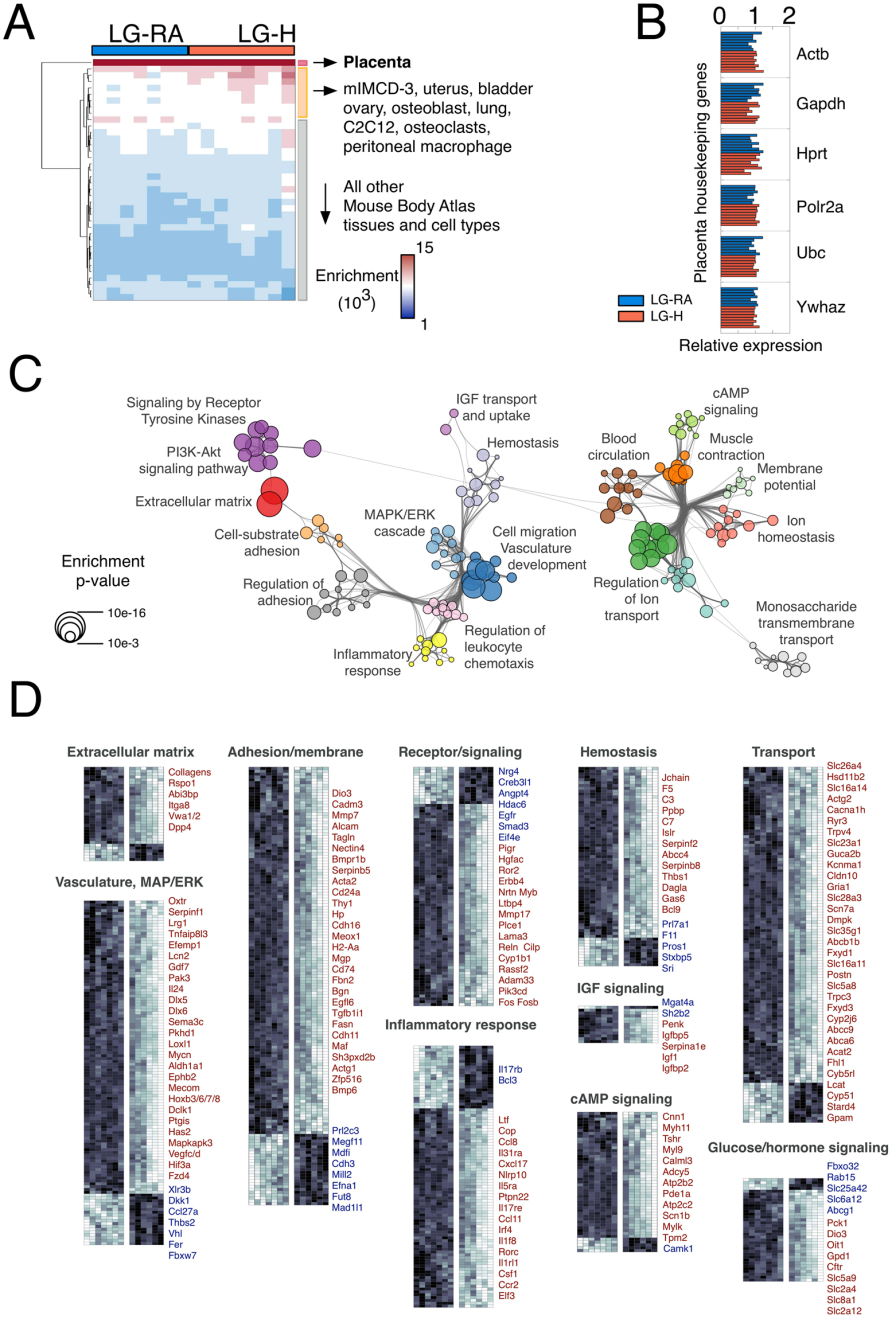
Late-gestational hypoxia induces significant changes in the placental transcriptome. (**A**) Hierarchical clustering of *SaVanT* enrichment scores for all the gene expression samples analyzed in this study (LG-RA n = 7, LG-H n = 8). Scores using all the expression signatures from the Mouse MOE430 Gene Atlas (BioGPS) were computed. Maximal enrichment was found for the placenta signature for all samples. Results for all tissues in the signature database are provided in Supplementary Table 2. (**B**) Relative expression profiles (using the average across all samples as reference) of placenta housekeeping genes (from Solano *et al*. 2016). (**C**) Network of significant functional categories for all genes differentially expressed between LG-RA and LG-H samples. Individual gene ontology terms with similar gene members are grouped by categories (node color), and labeled using a representative member. Node size is proportional to statistical significance. Edge thickness is proportional to between-node similarity and reflects the overlap between the gene sets annotated in both ontology terms. Only edges representing a Kappa similarity score greater than 0.3 are shown. Only significant ontology terms are shown (hypergeometric adjusted p-value p < 0.001). (**D**) Hierarchical clustering of expression profiles for genes grouped in broad functional categories from (**C**). Data for all the gene expression samples analyzed in this study is shown. Gene name colors indicate the direction of regulation (red: higher expression in LG-H, blue: lower expression in LG-H). Only representative genes in each category are labeled. Full results are provided in Supplementary Table 2. Genes annotated in several categories are only shown in one of them for clarity.

We performed whole-transcriptome RNA sequencing (RNA-Seq) on whole placentas isolated from LG-RA (n = 7) and LG-H (n = 8) mice at GD19. We first compared our data with a reference gene expression dataset comprising 91 mouse tissues (Mouse Gene Atlas, see Methods). All our samples were consistently enriched in the placenta expression signature, with minimal enrichment in other tissues (Fig. 2A and Supplementary Table 2). We next examined the expression profile of housekeeping genes in the mouse placenta, which were shown to be uniformly expressed across different gestational ages and conditions^22^. These genes showed marginal variability in expression for both LG-RA and LG-H samples (Fig. 2B). Taken together, these observations validate the integrity of the tissue profiled in this study and provide the basis for further differential analysis.

Next, we identified a set of 512 differentially expressed genes between LG-RA and LG-H placentas (Wald test adjusted p-value < 0.001, Supplementary Table 1). Ontology analysis revealed a functional network that allowed us to classify hypoxia-responsive genes into two largely distinct clusters (Fig. 2C, Supplementary Table 2). The first cluster comprised genes involved in tissue structure (including vasculature development, hemostasis, adhesion and extracellular matrix), and in receptor and intracellular signaling (i.e. MAPK, IGF, PI3K-Akt and tyrosine receptor pathways), including genes associated with inflammatory responses. The second cluster resulted from the enrichment of genes annotated in several nutrient and metabolic transport systems. For all functions, a majority of genes were found to be up regulated (higher expression in LG-H as compared to LG-RA) by hypoxia in late gestational placentas (Fig. 2D).

**Figure 3:**
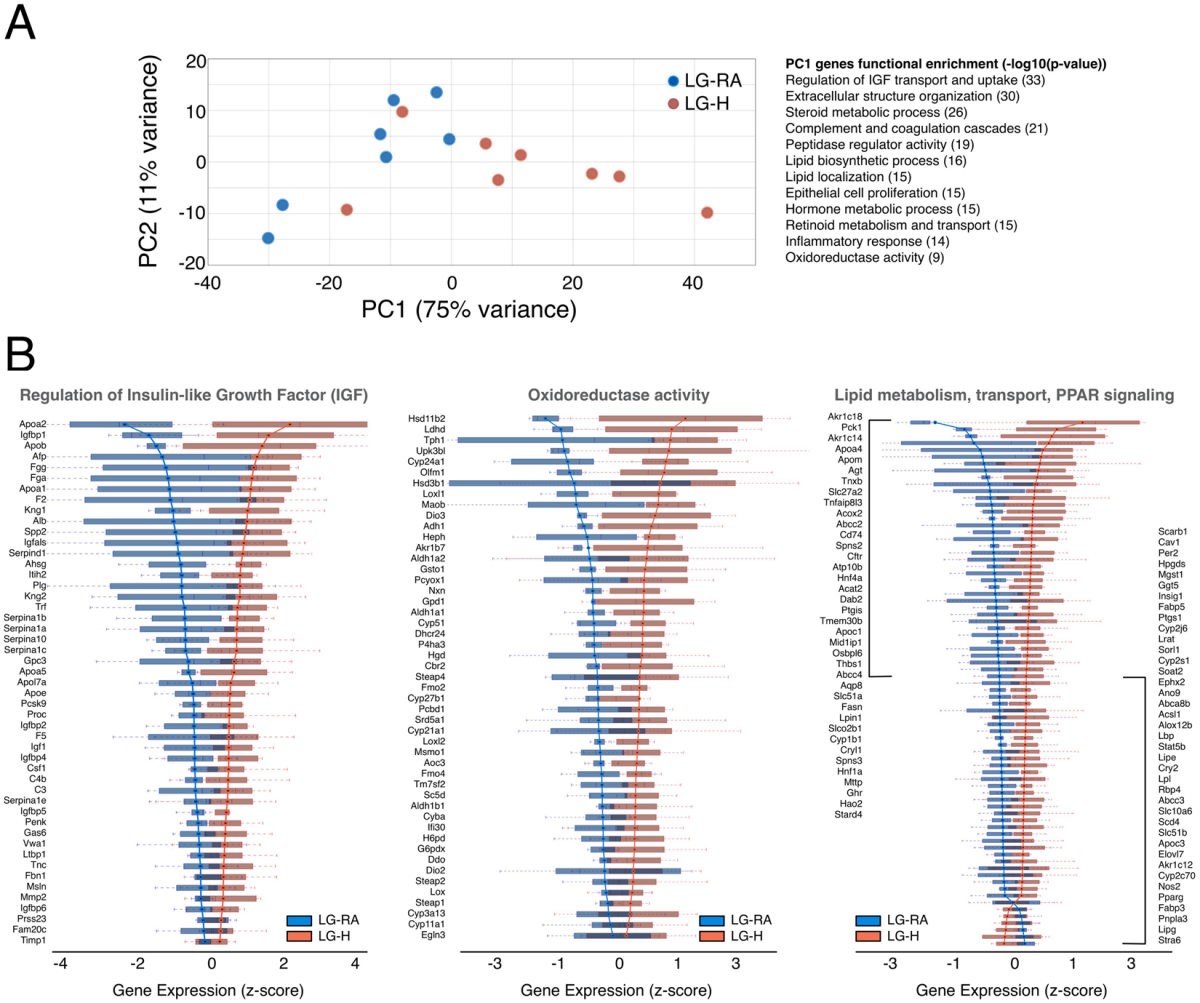
Principal component analysis of hypoxia-induced gene expression shifts. (**A**) Left: Principal component analysis of gene expression for LG-RA and LG-H placenta samples. Shown is the ordination of all samples using the first two principal components (PC1 and PC2). The ordination and percent of variance explained by each principal component was computed using whole-transcriptome expression levels. Right: functional enrichment results for the top 1000 genes ranked by PC1 loadings. Shown are the most significant ontology terms and enrichment scores (−log10 hypergeometric p-value). (**B**) Gene-wise boxplots of expression z-score distributions for genes annotated in selected functions from (**A**). Each box represents a gene’s z-score distribution for all samples in one experimental group (LG-RA n = 7, LG-H n = 8). Line plots connect the mean z-score for all genes in each experimental group. Official symbols for all genes in each functional category are shown.

Closer inspection of the expression profiles of hypoxia-induced genes showed that, although up-regulation was consistent across all our LG-H samples, many genes displayed a continuum in their response levels to hypoxia (Fig. 2D, Supplementary Table 1). In fact, principal component analysis (PCA) using the expression levels of all mouse genes provided a clear but continuous segregation between LG-RA and LG-H samples. This consistent but graded response to hypoxia was the dominant source of gene expression variability (PC1, first principal component, Fig. 3A), and accounted for 75% of the total variance in our whole-transcriptome dataset. We further analyzed the set of genes more strongly correlated with PC1 (see Methods). Strikingly, ontology analysis resulted in a highly significant enrichment for genes involved in the regulation of insulin transport (hypergeometric test p-value < 10e-33). This set of responsive genes was also significantly associated with steroid and lipid metabolism and transport, inflammatory response and oxidoreductase activity (Fig. 3A and Supplementary Table 2). The continuous but distinct distribution of expression levels of these genes in LG-RA and LG-H samples (Fig. 3B) suggested a varying yet strong shift in the activity of these pathways under late gestational hypoxia.

### RNA sequencing validation by qRT-PCR

RNA sequencing results were validated by real time polymerase chain reaction (RT-PCR). Four genes were chosen for validation –*Dio3*, *Hif3α*, *Pck1*, *Hsd11b2*–as these genes represent several of the pathways that were strongly affected in the placenta by gestational hypoxia exposure. All four genes demonstrated strong up-regulation in the LG-H groups compared to LG-RA, consistent with RNA sequencing results. For *Dio3*, relative gene expression calculated by comparative C_T_ method was increased ~30 fold (LG-RA: 1.11 ± 0.51, LG-H: 33.77 ± 35.9, p = 0.03 by Student’s t-test). For *Hif3α*, relative expression was increased ~2 fold in the LG-H group (LG-RA: 1.13 ± 0.67, LG-H: 2.43 ± 1.34, p = 0.04 by Student’s t-test). For *Pck1*, relative expression was increased ~75-fold in the LG-H group (LG-RA: 1.55 ± 1.41, LG-H: 118.21 ± 81.53, p = 0.002 by Student’s t-test). For *Hsd11b2*, relative expression was increased ~20 fold in the LG-H group (LG-RA: 1.19 ± 0.73, LG-H: 24.29 ± 23.20, p = 0.02 by Student’s t-test).

### Postnatal weight and milk intake studies

**Figure 4:**
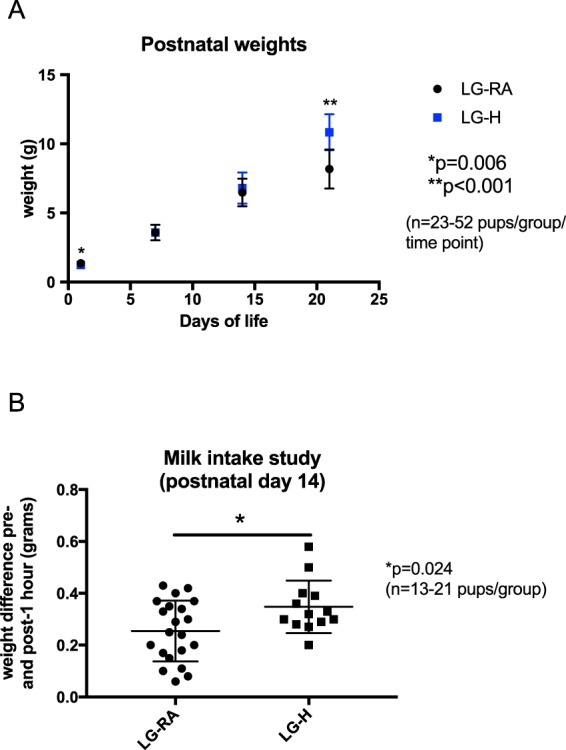
Postnatal weights in the first four weeks of life and milk intake studies in LG-RA and LG-H groups. Data are represented in graphs as means ± SD. (**A**) LG-H exposure results in decreased birthweight but increased weight at three weeks of postnatal age compared to LG-RA. The single asterisk indicates a significant difference between LG-H (blue) and LG-RA (black) groups (p = 0.006 by Student’s t-test) at birth, and the double asterisk indicates a significant difference between LG-H (blue) and LG-RA (black) groups (p < 0.001 by Student’s t-test) at three weeks postnatal age. (**B**) Milk intake, estimated as a weight change after one hour of suckling with the mother after a 5 hour fasting period, was ~25% greater in the litter size-matched LG-H group compared to the LG-RA group (p = 0.024 by Student’s t-test, denoted by asterisk).

Pup weights taken at 7, 14, and 21 days postnatal age were significantly different between groups at 21 days only (adjusted p-value < 0.001 by Holm-Sidak method) (Fig. 4A). This significant increase in weight in LG-H pups could be explained by an observed ~25% increase in milk intake as extrapolated from weight differences pre- and post- suckling for one hour measured on postnatal day 14 (p = 0.024 by Student’s t-test), even though litter sizes were matched (Fig. 4B).

### Body and organ weights and organ morphology with aging

**Figure 5:**
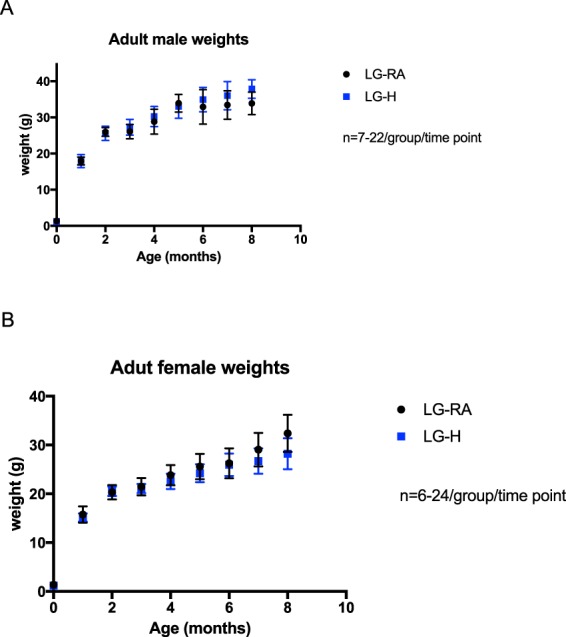
Adult weights for LG-RA and LG-H male and female offspring. There is not a significant difference between adult weights measured through 8 months of age, for LG-H males (blue squares) compared to LG-RA males (black) (**A**), or LG-H females (blue squares) compared to LG-RA females (black circles) (**B**). Data are represented in graphs as means ± SD.

For males and females exposed to hypoxia, there were no significant differences in adult body weights (p > 0.05 by Holm-Sidak testing for multiple comparisons). However, there was an interesting divergent trend by sex; adult male LG-H offspring were non-significantly heavier than male LG-RA offspring (Fig. 5A), while adult female LG-H offspring were non-significantly lighter than female LG-RA offspring (Fig. 5B) at older ages (eight months). There was not a difference in heart weight by hypoxia exposure between the four groups considered (p = 0.076 by two-way ANOVA for interaction; p = 0.0016 for sex; p = 0.6544 by hypoxia exposure). There was no difference in kidney weights between male or female LG-RA and LG-H groups (p = 0.42 by two-way ANOVA for interaction; p < 0.0001 for sex; p = 0.86 for hypoxia exposure). In males, the LG-H group exhibited a significant increase in abdominal fat content by weight, with an almost 2-fold increase in males (p = 0.0061 by Sidak’s multiple comparisons test), though an almost 3-fold increase in abdominal fat weight observed in females did not reach significance by two-way ANOVA testing (p = 0.0865 by Sidak’s multiple comparisons test) (p < 0.0001 by two-ANOVA for sex factor; p = 0.0007 by hypoxia exposure factor) (Table 2).

**Table 2:** Organ weights at 4 months of age in LG-RA and LG-H groups by sex.

Organ weights at 4 months of age (g)	Males (n = 8–13/group)		Females (n = 7–11/group)	
	LG-RA	LG-H	LG-RA	LG-H
Heart	0.125 ± 0.026	0.15 ± 0.024	0.108 ± 0.031	0.117 ± 0.023
Kidney	0.178 ± 0.030	0.184 ± 0.016	0.143 ± 0.015	0.134 ± 0.014
Abdominal Fat	0.649 ± 0.300	1.179 ± 0.409*	0.196 ± 0.093	0.563 ± 0.134

Data are expressed as mean ± SD. ^*^Indicates p < 0.05 by two-way ANOVA, between LG-RA and LG-H groups of each sex.

### Blood pressure with aging

**Figure 6:**
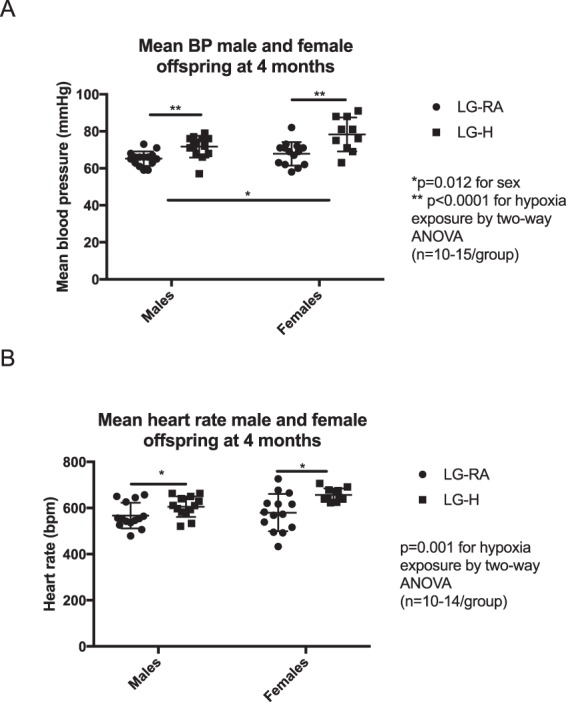
Mean blood pressure in LG-RA and LG-H male and female offspring at four months age. Data are represented in the graph as means ± SD. (**A**) LG-H males and females (squares) have higher mean blood pressures at four months of age than LG-RA males and females (circles) (p = 0.2944 for interaction, p = 0.012 for sex, p < 0.0001 for hypoxia exposure by two-way ANOVA, denoted by asterisk). (**B**) LG-H males and females (squares) have higher mean heart rates at four months of age than LG-RA males and females (circles) (p = 0.2532 by interaction, p = 0.059 by sex, and p = 0.0010 by hypoxia exposure by two-way ANOVA, denoted by asterisk).

We also analyzed the blood pressure of adult animals. At four months of age, both LG-H male and female offspring exhibited a 10–15% increased mean blood pressure (p = 0.2944 for interaction, p = 0.012 for sex, p < 0.0001 for hypoxia exposure by two-way ANOVA) (Fig. 6A), as well as increased heart rate (p = 0.2532 by interaction, p = 0.059 by sex, and p = 0.0010 by hypoxia exposure by two-way ANOVA) (Fig. 6B), when compared to LG-RA groups. This increase in mean blood pressure was persistent at 6 months in males and females (p = 0.0064 by hypoxia exposure, p = 0.0853 by sex, p = 0.9979 for interaction by two-way ANOVA), though a difference in heart rate was not detected (p = 0.8335 for interaction, p = 0.146 by sex, p = 0.3319 by hypoxia exposure by two-way ANOVA) (Supplementary Fig. 1).

### Plasma leptin and cholesterol concentrations

**Figure 7:**
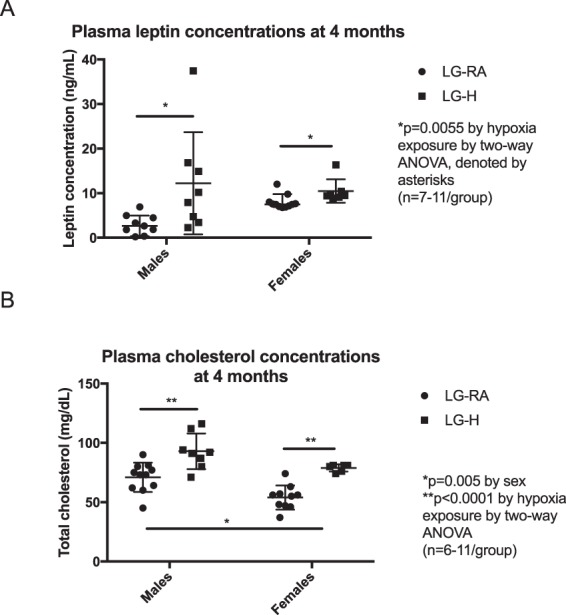
Plasma leptin and total cholesterol concentrations in LG-RA and LG-H male and female offspring at four months age. (**A**) In both males and females, LG-H groups had elevated plasma leptin concentrations compared to LG-RA groups (p = 0.0967 by interaction, p = 0.4091 by sex, p = 0.0055 by hypoxia exposure by two-way ANOVA, denoted by asterisks). Data are represented as median and 95% confidence intervals. (**B**) In both males and females, total cholesterol levels are elevated in LG-H offspring compared to LG-RA groups (p = 0.1637 by interaction, p = 0.0005 by sex, p < 0.0001 by hypoxia exposure by two-way ANOVA, significant values denoted by asterisks). Data are represented as means ± SD.

Given the changes in abdominal fat, total leptin and cholesterol concentrations were assessed in male and female LG-RA and LG-H groups at four months of age. In both males and females, LG-H groups had elevated leptin (p = 0.0967 by interaction, p = 0.4091 by sex, p = 0.0055 by hypoxia exposure by two-way ANOVA) (Fig. 7A) and total cholesterol concentrations (p = 0.1637 by interaction, p = 0.0005 by sex, p < 0.0001 by hypoxia exposure by two-way ANOVA) (Fig. 7B) compared to LG-RA groups.

## Discussion

Our animal study demonstrates that late gestational hypoxia exposure mimics placental insufficiency resulting in decreased birth weight and litter sizes, and moreover, an increased risk of cardiovascular disease in adulthood for the hypoxia-exposed offspring. Both male and female hypoxia-exposed offspring demonstrated evidence of hypertension, hypercholesterolemia, and increased leptin concentrations. There were sex-specific changes seen as well; male offspring exposed to prenatal hypoxia did not demonstrate differences in somatic weights compared to normoxia-exposed control males, but had significantly increased abdominal fat deposition. Hypoxia-exposed female offspring did not demonstrate significant increases in somatic or abdominal fat weights compared to normoxic controls. Importantly, we utilized this animal model to interrogate whether placental transcriptomic changes can serve as a surrogate for fetal genetic responses to in utero environmental exposures, and whether those changes specifically parallel alterations in cardiovascular and metabolic processes.

There are several existing murine models of placental insufficiency and IUGR, including maternal food restriction, uterine artery ligation, and maternal hypoxia exposure. Though poor nutrient provision and blood flow to the fetus are certainly implicated as important components of the IUGR condition, we chose to parse out and focus on chronic maternal hypoxia exposure as it mimics the common end point of poor oxygen delivery to the fetus thought to incur adverse fetal effects as seen in idiopathic human placental insufficiency/IUGR. Varying degrees of hypoxia, duration of hypoxia exposure, and timing of exposure all lead to differing effects on birth weight and litter size^9^. We selected the 10.5% oxygen levels from GD 14.5–18.5 in order to mimic human IUGR, which demonstrates lagging fetal growth predominantly in the third trimester. We found that this level of hypoxia, timing and duration of exposure not only generated growth-restricted pups, but also allowed for survival of the pups in order to study adult phenotypes. It should be noted that gestational hypoxia has been reported to decrease maternal food intake^23^, so it is likely that our findings are due to both a combination of hypoxia as well as nutrient restriction. We did observe decreased food intake (~60%) (measured in grams/gram of maternal body weight) in our hypoxia-exposed mothers, though this was measured in a small subset of mothers (2 mothers/group) and this observation was not powered to report a difference. Though we did find a difference in birth weight in offspring, we did not demonstrate a difference in fetal or placental weights at GD19, though the number of litters assessed at GD19 (n = 4–5 litters/group) was powered to detect difference by hypoxia exposure, but not by sex. Interestingly, our model allowed for observation of the natural “catch-up” growth phenomenon seen in human IUGR^24^. Even when controlling for litter size and therefore maternal milk availability, our hypoxia-exposed pups demonstrated increased milk intake and increased weight attrition postnatally during the suckling phase. Some studies utilize an altered diet postnatally^12^ in order to mimic conditions seen in Western countries and generate a phenotype of later cardiovascular disease. Our study supports the observation that this effect exists even without diet modification. “Catch-up growth” may be a persistent biologically programmed phenomenon after IUGR^24^ contributing to later adverse outcomes that should potentially be monitored closely, in order to protect against developmental programming of disease^25^.

Existing studies utilizing a similar gestational hypoxia exposure describe the effects of hypoxia on placental structure, function^26,27^, and specific pathways linked to cardiovascular disease^28^, though not on global transcriptomic changes. Other groups describe adult phenotypes of cardiometabolic and renal disease after hypoxia exposure^11,20,21^ in various wild-type and transgenic mouse models. Our findings are similar and complimentary to these previously published reports. The magnitude of our observed blood pressure differences between LG-RA and LG-H groups were similar to those reported by Walton *et al*.^20^, who utilized radiotelemetry in twelve month old mice to measure blood pressure. The consistency in results is reassuring, as we utilized tail cuff measurements which can be prone to error due to being operator dependent, requiring mouse acclimation/repeated training, and movement artifact^29,30^, which we minimized as much as possible. However, none of these studies link the two specifically – placental transcriptomic changes and adult outcomes. This linkage was a key objective in our study, given that the placenta, an organ often discarded after the delivery of a newborn, has both the identical genetic material as well as the same in-utero exposures as the fetus. Therefore, the study of the placenta potentially allows for non-invasive evaluation of fetal responses to adverse in-utero conditions, and can be mined for biomarkers that change as a response to the very processes that are mismatched pre- and post-natally, leading to developmental programming of cardiovascular disease.

In order to do this, our study utilized RNA sequencing to evaluate in a non-biased global manner, what the preferentially affected pathways were in the placenta after hypoxia exposure. Overall, our data revealed that both vascular and nutrient/metabolic transport pathways are strongly affected at the transcriptional level in our model of gestational hypoxia exposure (Fig. 2). Our differential expression results recapitulated the consistent dysregulation of genes previously associated with placental development and structure. For example, several members of the prolactin gene family (*Prl4a1*, *Prl7a1*, *Prl2c3*, *Prl2c2*) were downregulated in our models. These genes are coordinately involved on the response to physiological stress in the placenta^31^, and deficiency in Prl4a1has been linked to an impaired capacity of adaptation to hypoxia^32^. The parallelism between late gestational hypoxia and conditions associated with placental deficiency is further supported by the observed down-regulation of genes essential for vasculature and cardiovascular development such as *Mmp12*, *Vhl*, *F11*, *Fbxw7*, *and Bcl3*^32–36^. In the context of the signaling cascades induced by vascular injury, we also found a significant enrichment for genes involved in inflammatory response, and in particular, both *Cd74* and *Ccl8* are transcriptionally regulated in human pre-eclamptic placentas^37^. The consistent down-regulation of the heme oxygenase 1 gene (*Hmox1*) along with the upregulation of multiple serpin peptidase inhibitors (*Serpin1f* and *Serpinb5* among others) is in agreement with previous reports on the role of these hypoxia-related genes on placental development, IUGR and preeclampsia in humans and animal models^38,39^.

Gene expression analysis in our model also provided additional insights into the metabolic and homeostatic challenges induced by late gestational hypoxia. Of note, the most up-regulated gene (>16 fold) was phosphoenolpyruvate carboxykinase (*Pck1)*, which plays a major role on the maintenance of lipid metabolism, glucose homeostasis and insulin resistance^40^. Another example is a marked upregulation of the *adenylate cyclase 5* (*Adcy5*) gene, whose expression correlates with BMI, body fat mass, circulating leptin and fat distribution in both human and mice^41^. Fewer genes were found to be down-regulated in LG-H as compared to LG-RA placentas. However, these genes were also significantly associated with homeostatic processes. For instance, loss of function mutations on the *Mgat4a* gene are known to disrupt glucose and insulin homeostasis^42^, and this gene was found to be moderately but consistently downregulated in LG-H placentas. Finally, it is worth mentioning that classical members of the generic response to hypoxia including *Hif3a*, *Vegfc*, *Vegfd* were significantly induced in LG-H samples. As opposed to other members of the HIF and VEGF families, these less characterized genes are specifically associated with signaling pathways in adipose tissue. In fact, *Vegfc and Vegfd* are activators of lymphatic vessel growth and are involved in adipose tissue inflammation and obesity-related insulin resistance^43^. *Hif3a* was reported as the most significantly associated gene in a genome-wide study of DNA methylation and BMI^44^, and our up-regulation of this gene after hypoxia exposure parallels the role of *Hif3a* in the cellular response to glucose and insulin^45^. Combined, these examples underscore the departure from homeostatic equilibrium of the placenta under late gestational hypoxia.

Likely as a consequence of a disruption in placenta function and homeostasis was the dramatic upregulation in LG-H samples of multiple genes that regulate endocrine pathways. Among the most up-regulated genes was 11β-hydroxysteroid dehydrogenases 2 (*Hsd11b2*), which controls the amount of exposure to maternal glucocorticoids in the fetus^46^. *Hsd11b2* expression levels in the placenta has been shown to be dynamically regulated during cycles of acute and chronic stress, and its association with a long-term susceptibility of the offspring to neuroendocrine disorders is thought to be mediated by epigenetic mechanisms^47^. Consistent with our findings, alterations in *Hsd11b2* expression has been previously associated with other mouse models of growth restriction^27,48^. Another relevant example is the strong up-regulation of the hypoxia-inducible *Dio3* gene, an imprinted gene that is involved in thyroid hormone metabolism, potentially indirectly influencing fetal exposure to thyroid hormones. Alterations in fetal thyroid hormone concentrations have been suggested to play a role in programmed postnatal disease^49^. It is therefore tempting to speculate that the observed upregulation of these genes is a fingerprint of a generic stress-sensing mechanism in the placenta, with a potential role in the onset of developmental programming.

The previous results highlight the complex functional interactions that are integrated and tightly regulated in the placenta. The phenotypes in both the placenta and the offspring that result from our model will most likely be influenced by additional factors (e.g. diet, food intake and sex, among others). One could therefore anticipate that the transcriptional response to hypoxia will also be modulated by those extrinsic factors^7^. In fact, beyond the consistency imposed by our stringent statistical thresholds, we observed that many of the gene expression changes between LG-RA and LG-H samples discussed above were not discrete, with specific samples showing a more dramatic transcriptional response. However, further analyses revealed that this gradual shift in expression across different hypoxic placentas was not accidental. On the contrary, it was remarkably correlated with a shift in the activity of the regulation of insulin and lipid transport and metabolism, along with a marked enrichment of protective genes. Therefore, our transcriptional data support the results from recent reports on the role of the insulin-IGF pathway on the integration of nutrient and oxygen signals in the placenta^23^, and the role that multiple transport and endocrine systems might have in these processes^50,51^, as discussed above. The role that the specific transcriptional changes found in our model might have on the onset of developmental programming warrants additional studies. In this respect, it will be interesting to analyze the role of genes previously associated with epigenetic modifications during pregnancy like *Mfap4*, *Hif3a*, *Nnat* and *Pmaip1*^52,53^, as they were strongly correlated with the hypoxia-induced shifts reported here.

The fact that significant sex-specific differences in the placental transcriptome are not reported here could be attributed to the small sample size. Also, sex-specific biologic programming of responses to in-utero stress may be more postnatally mediated. Consistent with several animal studies^11,20,21,54,55^ that suggest that while all offspring are at increased risk of later disease, we found that males are more susceptible to risk of developmental programming of cardiovascular disease after various in utero insults. We found in our model that both adult males and females demonstrate elevated blood pressure, total cholesterol levels, and elevated leptin, but males demonstrated tendency towards obesity with increased abdominal fat deposition, whereas females do not demonstrate tendency to obesity after hypoxia exposure in utero. The development of these adverse outcomes in females that are not outwardly apparent in overall body weight, suggests that cardiovascular risk may be “overlooked” in females. It is tantalizing to hypothesize that these adverse effects may become exaggerated and even “unmasked” in pregnancy, where rapid weight gain and insulin resistance are physiologic, perhaps contributing towards gestational hypertension and diabetes conditions, as has been seen previously^56^.

Lastly, we probed our RNA-sequencing data for biologic plausibility and comparability to the human condition of placental insufficiency. In humans, placental insufficiency has been linked to recurrent miscarriage, preterm birth, pre-eclampsia as well as IUGR. As discussed previously, a number of the differentially expressed genes in our LG-H group have been shown to be associated with pre-eclampsia in human cohorts^33,57^. A recent large genome-wide association study in humans reported a handful of genes – EBF1, EEFSEC, AGTR2, WNT4, ADCY5, and RAP2C - that were significantly associated with length of gestational or risk of delivery prior to 37 weeks’ gestation^58^ in a genetically homogeneous population. Of the six gene variants, three of these genes – EBF1, AGTR2, ADCY5 - are differentially expressed in our murine model of gestational hypoxia exposure, potentially implicating pathways upregulated in hypoxia-induced stress in the condition of premature birth.

We present here the first study to specifically link the placental transcriptome to risk of developing adult cardiovascular disease in a murine model of IUGR caused by late gestational hypoxia exposure. This *in vivo* model of developmental programming of CVD allows for future evaluation of the utility of the placental genome and epigenome as a reflection of both the genetic and prenatal environmental influences seen by the fetus. This model can be used in future studies to probe for epigenetic mechanisms linking in utero stressors to developmental programming of adult CVD in a sex-specific manner, as well as to potentially test future prevention modalities and intervention strategies against the developmental programming of disease in the IUGR murine offspring.

## Methods and Materials

### Ethical Approval

This study was conducted per established guidelines and all protocols were approved by the Animal Research Committee of the University of California Los Angeles in accordance with the National Institutes of Health. C57BL/6 mice obtained from the UCLA Division of Laboratory and Animal Medicine colony (RRID:IMSR_JAX:000664) were housed in 12:12 hour light-dark cycles with *ad libitum* access to a standard rodent chow diet (Pico Lab Rodent Diet 20, cat# 5053, Lab Diet, St. Louis, MO) and water. At the end of the experimental period, mice underwent brief anesthesia using inhaled isofluorane (2–3 minutes) followed by cervical dislocation. The tissues of interest were dissected within 5 minutes of euthanasia, and immediately stored for downstream processing (quick frozen in liquid nitrogen and stored at −80 °C until RNA extraction). All efforts were made to reduce the number of animals used for experiments, and to minimize animal suffering. The investigations presented here comply with the animal ethics policy and regulations set out in the editorial, abiding with the principles under which this journal operates.

### Placental samples

At 2–3 months of age, male and female mice were mated overnight, and pregnant female mice were identified by the presence of a vaginal plug the following morning (designed as gestational *day 0*.*5*). Pregnant females were transferred to individual cages and reared on the same chow diet ad libitum until gestational *day 14*.*5*, at which point they were randomly assigned to two groups: 1) late gestation-room air (LG-RA) pregnant mice which were housed in standard conditions of 0.21 FiO_2_, and 2) late gestation-hypoxia (LG-H) pregnant mice were exposed to hypoxia (0.105 FiO_2_) for 4 days (days 14.5 to 18.5 of gestation). Hypoxia exposure consisted of 10.5% fractional inspired oxygen concentrations continuously, which was maintained using a commercially available environmental system (Biospherix ProOx model P360, Parish, NY, USA). For placental studies, on *GD 19*, animals were euthanized by brief exposure to isofluorane followed by cervical dislocation. Under laparotomy followed by hysterotomy, the placentas inclusive of the maternal decidua, were separated from the respective fetuses and collected (n = 7–30 placentas/group, arising from 5 litters for LG-RA conditions and 4 litters from LG-H conditions). The placentas and fetuses were weighed in a Mettler AB104 precision balance (0.01 mg sensitivity), and then immediately snap-frozen and stored at −80 °C until further analysis. For offspring studies, pregnant females were removed from the chamber and allowed to deliver pups normally, and measurements taken accordingly (for birth weight, litter size, genotyping measurements, n = 7 litters for LG-RA and LG-H groups).

### Genotyping

DNA was isolated from ~0.2 cm tail samples using the Bioland Quick Genotyping DNA preparation kit (Bioland Scientific, Paramount, CA, USA), according to the manufacturer’s protocol. DNA was quality checked using Nanodrop prior to genotyping. Genotyping was performed on ~50 ng of DNA per sample via PCR using standard primers for genetic sex determination in mice (B451: Forward primer, 5′-GATGATTTGAGTGGAAATGTGAGGTA-3′; B452: Reverse primer, 5′-CTTATGTTTATAGGCATGCACCATGTA-3′)^59^ and Dream Taq hot start green master mix (Thermo Scientific, cat#K9021, Waltham, MA, USA). Primers were brought to a working concentration of 0.4 µM. PCR conditions were as follows: denaturing at 95° for 2 minutes, followed by 35 cycles of the following–denaturation at 95 °C for 30 seconds, annealing at 59 °C for 30 seconds, and extension at 72 °C for 60 seconds. This was followed by a final extension at 72 °C for 5 minutes. Samples were then visualized on a 1.2% agarose gel with green safe gel stain (Bioland Scientific, cat# SAFE01-01, Paramount, CA, USA). Males were verified with Y chromosome visualization at band length 280 bp, while females demonstrated bands at 480 bp (weak) and 660 bp (strong).

### RNA sequencing libraries and data analysis

Sample sizes for RNA sequencing were based upon previously published data evaluating placental gene expression using a murine model of maternal food restriction resulting in IUGR^7^. Power analyses conducted using gene expression data (by qRT-PCR) from that study, which showed mean relative expression = 1.0 with SD = 0.1, indicate that 5–8 animals per experimental group can detect a difference of at least 25–35% using the Hsu (with Best) multiple comparison test with 89% power at a 0.05 overall significance level.

Frozen whole placental tissues, including the maternal decidua, obtained from LG-RA and LG-H groups (n = 7–8/group from 7 litters for LG-RA and from 4 litters for LG-H) were employed. Briefly, tissue from single placentas was homogenized and RNA extracted using the Zymo Direct-Zol RNA MiniPrep Plus (Zymo, cat# R2070, Irvine, CA) as per the manufacturer’s instructions. Total RNA was quantified and 260/280 ratios determined using Nanodrop (all samples with a 260/280 = >1.97). One μg of total RNA was used as starting material for each sample. RNA sequencing and library preparation was performed by the UCLA Technology Center for Genomics and Bioinformatics using the KAPA Stranded mRNA-seq kit (Roche Sequencing, cat#KK8421, Pleasanton, CA, USA), according to the manufacturer’s instructions. The work flow consisted of mRNA enrichment, cDNA generation, end repair to generate blunt ends, A-tailing, adaptor ligation and PCR amplification. Different adaptors were used for multiplexing samples in one lane. Sequencing was performed on the HiSeq. 3000 System for a single-read 50 run (Illumina). The raw data has been deposited into the NCBI’s Gene Expression Omnibus (GSE112755).

Data quality check was done on an Illumina SAV. Demultiplexing was performed with the Illumina Bcl2fastq2 v 2.17 program. The STAR ultrafast universal RNA-seq aligner v2.5.2b^60^ was used to generate the genome index and to perform paired-end alignments. Reads were aligned to a genome index that includes both the genome sequence (GRCm38 primary assembly) and the exon/intron structure of known gene models (Gencode M12 genome annotation). Alignment files were used to generate strand-specific, gene-level count summaries with STAR’s built-in gene counter. Only protein-coding genes in the Gencode M12 annotation were considered (95% of total counts on average). Independent filtering was applied as follows: genes with less than 15 counts across all samples, count outliers or low mappability (<100 bp) were filtered out for downstream analysis^61,62^. Expression estimates were computed in units of fragments per kilobase of mappable length and million counts (FPKMs). The table of expression estimates (FPKM) was used as input for *SaVanT*^63^ to compute enrichment scores on mouse gene expression signatures (Mouse MOE430 Gene Atlas (http://biogps.org/)) and relative expression profiles. Non-default parameters for *SaVanT* were “*Convert matrix values to ranks*” and “*Compute null distribution with 10000 iterations*”. Differential expression analysis was performed with *DESeq*. *2* (Bioconductor, v3.7, RRID:SCR_015687)^62^. Count data was fitted to additive models using *Sex* (female/male) and *experimental group* (LG-RA/LG-H) as explanatory factors. The individual effect of each factor on the expression of each gene was tested using a contrast with reduced models (likelihood ratio test). Additional models including a Sex:Group interaction term were used to fit the data and identify genes with a sex-specific response to hypoxia. Pairwise differential expression was performed to classify genes as differentially expressed (Wald adjusted p-value < 0.001) between LG-RA and LG-H placentas (512 genes). Unfiltered differential expression results are provided in Supplementary Table S2. Functional enrichment was performed with Metascape (http://metascape.org) and visualized in Cytoscape (Cytoscape, v3.6.1, RRID:SCR_003032)^64^. Principal component analysis (PCA) was performed in R (R project for statistical Computing, v 3.4.4, RRID:SCR_001905) on variance-stabilized expression data from DESEq. 2. Genes with high PC1 loadings were further analyzed with Metascape, and distributions of variance-stabilized expression z-scores for genes in specific functional categories was visualized as box plots. With the exception of Fig. 2B, all figures were generated in Matlab (MATLAB, version release 2017a, The MathWorks, Inc, RRID:SCR_001622).

### RNA sequencing validation by real time quantitative PCR (RT-PCR)

For RT-PCR analysis, total cellular RNAs were isolated using the RNeasy mini kit (Qiagen, cat #74104, Valencia, CA. USA) according to the manufacturers’ instructions. Quantitative and qualitative analyses of isolated RNA were assessed by the ratio of absorbance at 260 and 280 nm. cDNA was generated from 1 μg of total RNA from placental tissue using reverse transcription (RT) using a Superscript III Reverse Transcriptase kit (Invitrogen, cat#18080093, San Diego, CA, USA) following the manufacturer’s instructions. The reverse transcription was performed at 50 °C using 5 μg/μl of random hexamers. Real-time PCR amplification was performed in triplicate using Taqman-based detection according to the manufacturer’s instructions on a Step One real-time quantitative PCR thermocycler (Applied Biosystems, Foster City, CA, USA). Four genes with clinical relevance to the pathways most affected by gestational hypoxia exposure by RNA sequencing results were chosen for validation: *Dio3*, *Hif3α*, *Pck1*, and *Hsd11b2*. Commercially available Taqman gene expression assay kits were used to quantitate gene expression (Thermofisher, cat#4448892 and 4453329, Waltham, MA, USA). Relative gene expression was calculated using the comparative C_T_ method with 18 S (Applied Biosystems, #4319413E, Foster City, CA, USA) expression used as the internal control for normalization. The amplification cycles consisted of: 50 °C for 2 minutes, 95 °C for 20 seconds, then 45 cycles of 95 °C for 1 second (denaturation), annealing for 20 seconds.

### Offspring birthweights, litter counts and adult body and organ weights

Given the variable effects on pup viability after hypoxia exposure, litter numbers were counted within 12 hours of delivery and the number of viable versus stillborn pups determined at that time. Birthweight was also obtained at that time. Mothers were maintained on the standard rodent chow diet throughout pregnancy and during lactation (Pico Lab Rodent Diet 20, cat# 5053, Lab Diet, St. Louis, MO). Pups were weighed at 7, 14, and 21 days (n = 23–52 pups/group/time point from 7 litters each for LG-RA and LG-H groups). Pups were weaned from their nursing dams at 21 days after birth, and males and females were caged separately. Offspring were maintained on the same standard rodent chow diet (Pico Lab Rodent Diet 20, cat# 5053, Lab Diet, St. Louis, MO). Offspring were then weighed at 4, 8, 12, 16, 20, 24, 28, and 32 weeks of age (n = 6–24/group/time point for males and females separately). In an arbitrarily selected subset of mice (n = 6 litters for LG-RA and 3 litters for LG-H), at 4 months of age, mice were euthanized with isofluorane and blood was collected after cervical dislocation. Organs of interest – heart, kidneys, and abdominal fat–were also collected and weighed, then snap frozen in liquid nitrogen and stored at -80 °C until further processing (n = 8–13/group for males, and n = 7–11/group for females).

### Milk intake studies

Milk intake studies were performed on litters matched for size (litters that were naturally born of similar numbers were examined; n = 3 LG-RA litters, 2 LG-H litters), such that each litter considered had 6–7 pups/litter. Fourteen day-old suckling pups were isolated from their mothers in the morning and maintained in their cages on a heated pad for five hours. They were then placed back with their mothers for suckling. They were individually weighed at the time of separation, just prior to replacement with the mothers, and then one hour after suckling with their mothers as described above. The difference in pup weights (mg) was used as a surrogate for milk intake (ml)^65^.

### Blood pressure tail-cuff measurements

At four months of age, blood pressure measurements of males and females from both groups (taken from n = 5 litters for LG-RA and 7 litters for LG-H) were obtained using tail-cuff blood pressure measurements taken using the BP2000 Series II Blood Pressure Analysis System (Visitech Systems, Apex, NC, USA). To perform these studies, mice were restrained inside a container placed on top of a heated platform. The mouse tail was placed into a tail blood pressure cuff and tail blood pressure measurements were taken every two minutes for twenty minutes to allow for an acclimation period (n = 13–15/group for males, n = 9–17/group for females). Blood pressures were taken at the same time on various days for all mice, and measurements were performed in a room where minimal noise could be assured.

### Plasma leptin and cholesterol levels

Plasma was collected by obtaining whole blood after euthanasia of mice at four months of age (n = 6 litters for LG-RA and 3 litters for LG-H). Whole blood was collected into EDTA tubes (BD, Franklin Lakes, NJ, USA) and then centrifuged at 8,000 rpm for 10 minutes. The top layer of plasma was pipetted into a separate EDTA tube and all samples were stored at − 80 °C (n = 8–9/group for males, n = 7–11/group for females). Leptin concentrations were measured using a mouse leptin ELISA kit (Thermo Fisher Scientific, cat#KMC2281, Waltham, MA), according to the manufacturer’s instructions, at a dilution of 1:32 for males and 1:16 for females. Standard curves were constructed with R values of 0.95–0.99, and concentrations (in ng/mL) were determined based upon the standard curve. Values outside the range of the standard curve were excluded from analysis. For the leptin assay, the appropriate range was 93.8–6000 pg/mL, with the sensitivity threshold at 50 pg/mL, and intra- and interassay variations at 2.75% and 3.54%, respectively, within the assay range, and coefficient of variation 2.46.

Total cholesterol concentrations were measured on 10 μL plasma samples obtained from mice (n = 8 = 11/group for males, n = 6–10/group for females). These samples were processed by the UCLA Division of Laboratory Animal Medicine, using the ACE Cholesterol reagent kit (Alfa Wassermann, ref #SA1010, West Caldwell, NJ, USA), with a reported sensitivity of detection between the concentration range of 6–600 mg/dL (correlation coefficient = 0.9990).

### Statistical Analysis

All statistical analyses described in sections 2.2 were conducted in GraphPad Prism software (version 5, GraphPad Software Inc., La Jolla, CA) and data are presented as means ± SD when data were normally distributed, and as median and quartile ranges or 95% confidence intervals provided when not normally distributed, unless otherwise indicated. Birthweights were normally distributed, as has been previously reported in C57BL/6 mice^66^, but litter sizes were not^67^, using the D’Agostino and Pearson normality test. Somatic weight at 4 weeks of age in male mice was normally distributed, so we assumed normal distribution for all weight data sets subsequently in males and females. Normality was also tested using the data obtained from control LG-RA groups for other tests (blood pressure, cholesterol, leptin, milk intake). If the sample size was too small to test for normality, we assumed data were not normally distributed (e.g. sex-specific placental and fetal weight differences in the LG-H group, and gestation length). All data sets with adequate sample size, except for leptin concentrations, demonstrated a normal distribution. To test for significant differences between two groups (LG-RA- and LG-H) for normally distributed data (e.g. birthweight data, milk intake, qRT-PCR), we analyzed results using the Student’s t-test for un-paired parametric data. To compare un-paired data that demonstrated a skewed distribution (litter sizes), Mann-Whitney U test was used. For body weights over time (at one month increments), multiple comparison testing was employed using the Holm-Sidak method. For multiple comparison groups (e.g. organ weights, blood pressure, cholesterol and leptin concentrations), to analyze the effect of sex and treatment, two-way ANOVA testing was performed and Sidak’s multiple comparison testing used to distinguish hypoxia-exposure effect by sex. All p-values are reported as 2-tailed with statistical significance set at <0.05 for all comparisons.

Sample sizes for physiologic blood pressure were based upon previously published data evaluating adult mouse blood pressures after postnatal hypoxia exposure in the mouse^68^. Power analyses conducted using blood pressure data from that study, showing mean systolic blood pressure = 115, with SD = 5.5, indicate that 8 animals per group was sufficient to detect a 10 mmHg change in blood pressure, achieving >99% power with an overall significance level set at 0.05.

Sample sizes for plasma leptin and cholesterol concentrations were based upon previously published data evaluating plasma leptin concentrations after intermittent gestational hypoxia exposure in the mouse^11^. Power analyses conducted using reported leptin concentrations from that study, showing mean plasma leptin concentrations of 2.8 ng/mL with SD = 0.83, indicate that 7–10 animals per group was sufficient to detect a 25% change in leptin concentrations, achieved an ~80% power with overall significant level set at 0.05.

## Supplementary information

Figure S1

Table S1

Table S2

## Data Availability

All data generated or analyzed during this study are included in this published article (and its Supplementary Information files).
