# Supplementary material for: The Placental Transcriptome in Late Gestational Hypoxia Resulting in Murine Intrauterine Growth Restriction Parallels Increased Risk of Adult Cardiometabolic Disease
Source: Sci Rep. 2019 Feb 4;9:1243. doi: 10.1038/s41598-018-37627-y (PMC6361888; doi:10.1038/s41598-018-37627-y)
Supplement: Supplementary file 1 — Figure S1 [file 41598_2018_37627_MOESM1_ESM.pdf]

THE PLACENTAL TRANSCRIPTOME IN LATE GESTATIONAL HYPOXIA RESULTING IN MURINE INTRAUTERINE GROWTH RESTRICTION PARALLELS INCREASED RISK OF ADULT CARDIOMETABOLIC DISEASE

Alison Chu, MD[1a*], David Casero, PhD[2], Shanthie Thamotharan, MS[1b], Madhuri Wadehra, PhD[3], Amy Cosi, MS[1c], Sherin U. Devaskar, MD[1d]

[1]David Geffen School of Medicine at UCLA, Department of Pediatrics, Division of Neonatology & Developmental Biology, Neonatal Research Center of the UCLA Children's Discovery and Innovation Institute. 10833 Le Conte Avenue, MDCC B2-375, Los Angeles, CA, 90095, USA; [a]alisonchu@mednet.ucla.edu; [b]sthamotharan@mednet.ucla.edu; [c]olsonamyn@ucla.edu; [d]sdevaskar@mednet.ucla.edu

[2]David Geffen School of Medicine at UCLA, Department of Pathology and Laboratory Medicine, 3000 Terasaki Life Sciences Building, 610 Charles Young Drive East, Los Angeles, CA 90095, USA; dcasero@g.ucla.edu

[3]David Geffen School of Medicine at UCLA, Department of Pathology and Laboratory Medicine, 4525 MacDonald Research Laboratories, Los Angeles, CA 90095, USA; mwadehra@mednet.ucla.edu

*Corresponding author:

Alison Chu, David Geffen School of Medicine at UCLA, Department of Pediatrics, Division of Neonatology, 10833 Le Conte Avenue, MDCC B2-375, Los Angeles, CA 90095; (t) 310.825.9436, (f) 310.267.0154; alisonchu@mednet.ucla.edu

A

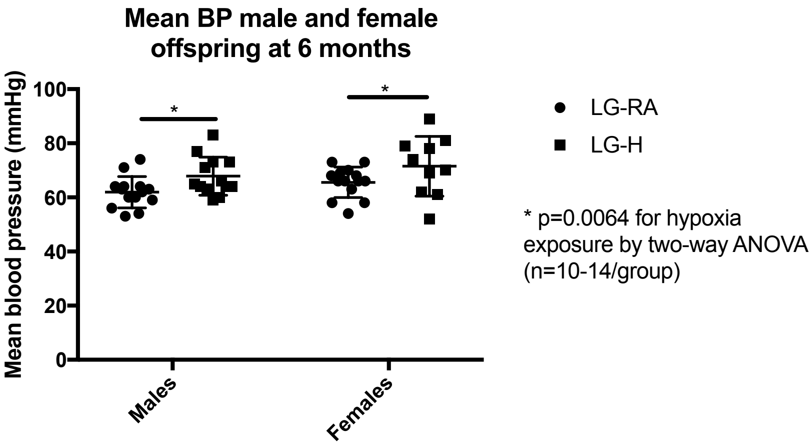

B

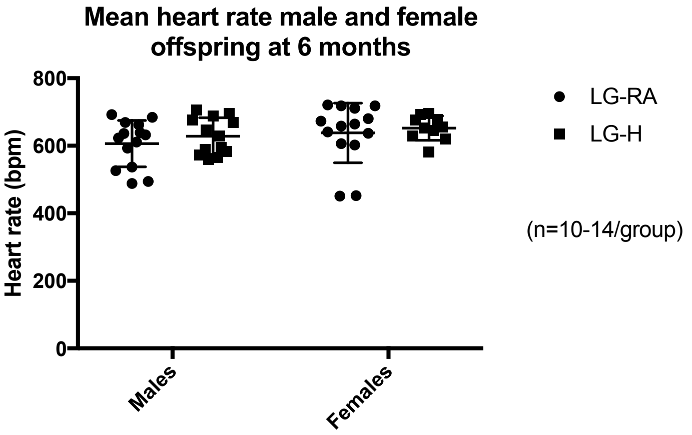

Supplemental Figure 1: Mean blood pressure and heart rate in LG-RA and LG-H male and female offspring at six months' age. Data are represented in the graphs as means±SD. (A) LG-H males and females (squares) have higher mean blood pressures at six months' age than LG-RA males and females (circles) ($p=0.9979$ for interaction, $p=0.0853$ by sex, $p=0.0064$ by hypoxia exposure by two-way ANOVA). (B) LG-H males and females (squares) do not demonstrate differences in mean heart rate compared to LG-RA males and females (circles) ($p=0.8335$ for interaction, $p=0.1460$ by sex, $p=0.3319$ by hypoxia exposure by two-way ANOVA).
